# Validation of an instrument to measure the perception of occupational safety and health among Peruvian dentists

**DOI:** 10.1038/s41598-025-00395-7

**Published:** 2025-05-02

**Authors:** Marysela Ladera-Castañeda, José Escobedo-Dios, Alberto Cornejo-Pinto, Jenny Cieza-Becerra, Miriam Castro-Rojas, Carlos López-Gurreonero, César Cayo-Rojas

**Affiliations:** 1 Faculty of Dentistry and Postgraduate School, Research Group “Salud Pública-Salud Integral”, Universidad Nacional Federico Villarreal, Lima, Peru; 2 School of Stomatology, Universidad Privada San Juan Bautista, Lima, Peru

**Keywords:** Health and safety, Occupational health, Occupational safety, Validation study, Health care, Health occupations

## Abstract

Dentists encounter a variety of occupational hazards in the practice of dentistry, with the potential to impact their general well-being and the quality of service provided to patients. This study aimed to validate an instrument for measuring the perception of occupational safety and health among Peruvian dentists. This was an instrumental study in which 379 Peruvian dentists participated. The instrument on the perception of occupational safety and health in dentists was adapted and validated using the NTP 182 (Self-assessment survey of working conditions) as a reference. Content validity was assessed by means of the Aiken V. The internal structure was assessed by exploratory factor analysis (EFA), principal component analysis (PCA) and confirmatory factor analysis (CFA). The internal consistency of the instrument was assessed with Cronbach’s alpha. The content analysis by expert judges supports the representativeness of the items related to the construct. Four dimensions were established by means of the EFA, PCA and CFA: work demands and well-being, ergonomics and physical conditions of the environment, safety and risk prevention, and working conditions and worker protection. Regarding the AFC, adequate fit indices were evidenced: Chi-square (χ^2^) = 321.071, degrees of freedom (df) = 206, χ^2^/df = 1.559 (*p* < 0.001), standardized root mean square residual (SRMR) = 0.047, comparative fit index (CFI) = 0.974, Tucker and Lewis index (TLI) = 0.963, weighted root mean square residual (WRMR) = 0.045 and root mean square error of approximation (RMSEA) = 0.038. Furthermore, the internal consistency of the questionnaire using Cronbach’s alpha was very good (α = 0.846). The simplified questionnaire to assess dentists’ perceptions of occupational safety and health has been demonstrated to be both valid and reliable. Its utilization for research purposes is recommended, with a focus on the following four dimensions: work demands and well-being, ergonomics and physical conditions of the environment, safety and risk prevention, and working conditions and worker protection. To ensure the validity of the findings, it is advised that the questionnaire be administered to a larger sample in a range of social and geographical contexts.

**Supplementary Information:**

The online version contains supplementary material available at 10.1038/s41598-025-00395-7.

## Introduction

Occupational Safety and Health (OSH) is considered an inalienable right of all workers whose purpose is to prevent accidents and occupational diseases in the workplace. Therefore, organizations must implement measures to enhance working conditions with a view to avoiding physical and psychological problems among workers^1^.

The International Labour Organization (ILO) reports that approximately 317 million people worldwide experience occupational accidents annually, with 2.34 million fatalities resulting from occupational accidents or diseases^2^. This shows that safety and health issues at work happen a lot, and they’re much worse in developing countries. This is because many workers can be physically and mentally hurt by being exposed to different risks at work, which can have personal, family, and social effects^3^.

However, significant progress has been made in the field of occupational safety and health due to the existence of laws, directives, decrees, and guidelines adopted by various countries to regulate the issue. Nevertheless, the absence of clearly delineated and standardized roles for diverse professional categories remains a salient concern^4^. The laws in Peru that cover health and safety at work are set out in Law No. 29,783 and the rules that go with it, which were made official by Decree No. 005-2012-TR. This legislative apparatus is applicable to all services and economic sectors, as well as to all employers and public servants nationwide^1^.

An occupational hazard is defined as an injury or ailment resulting from work or the work environment, which can result in trauma, post-traumatic stress disorder (PTSD), loss of dignity, anxiety, depression, suicide attempts, low self-esteem, lack of trust in people, aging, loss of autonomy, absenteeism, physical injuries, musculoskeletal disorders, among others^3,5,6^. Research indicates that the primary biological hazards to which health professionals are exposed are needlestick injuries, affecting 80% of workers, and exposure to contaminated substances, present in 75% of cases. With regard to non-biological risks, the most prevalent are back pain, affecting 79% of professionals, and overtime, affecting 72% of them^3,7^.

Occupational safety and health are pivotal to achieving the 2030 Agenda for Sustainable Development, as it relates directly to several of its goals, in particular the 3rd Sustainable Development Goal (SDG), which aims to reduce pollution-related mortality; the 8th SDG, which promotes labor rights in safe environments; and the 16th SDG, which calls for effective and transparent institutions^8^. These goals underscore the imperative to establish safe working conditions as an integral component of sustainable development^8^.

Dentistry is widely regarded as a high-risk profession. This is due to the exposure of dentists to a variety of harmful factors, including radiation, percutaneous exposure incidents, exposure to dental materials, noise and vibrations, as well as allergic problems, vision problems, musculoskeletal disorders, occupational violence, and sedentary work^9–12^. Furthermore, in comparison with other health professionals, dentists are in constant contact with patients and utilize high-speed rotating instruments, which generate contaminated bioaerosols and expose them to various infectious diseases^3,10,13^. Conversely, stress arising from interactions with patients, daily routine, and compliance with stringent healthcare procedures contributes to the development of psychological problems, thus classifying them as one of the most susceptible work groups in healthcare^3,10,13^. It is therefore vital to recognize, monitor, and properly manage occupational risks in order to mitigate their consequences^3,13^.

A literature review reveals the existence of some validated instruments related to occupational safety and health for dentists. One such instrument is the Interdisciplinary Worker Health Approach Instrument (IWHAI), which, as its name suggests, is interdisciplinary in approach and not exclusively designed for dentists, but rather for the general assessment of health aspects^14^. In contrast, Garcia’s study^15^ utilized the Nordic Workplace Safety Questionnaire to assess dental center workers’ perceptions of safety in their work environment. However, this questionnaire primarily focuses on employees’ perceptions of general safety management policies and practices, neglecting to address the specific risks and health-related aspects that are pertinent to dentists. Furthermore, the study by Ramaswami et al.^16^ utilized a validated questionnaire to evaluate knowledge regarding risks and preventive measures; however, it did not assess workers’ perceptions of safety and health in their workplace. Finally, the research by Reddy et al.^17^ employed a validated questionnaire to assess dentists’ perceptions of occupational hazards and preventive measures. Nevertheless, it did not encompass significant aspects of physical environmental conditions and work demands that may influence dentists’ health.

It is imperative that companies or organizations adhere to the prevailing protocols concerning occupational safety and health. The repercussions of occupational diseases and accidents on the lives of workers are manifold, encompassing not only human suffering for employees and their families but also substantial economic losses for organizations. These losses manifest in the form of elevated healthcare expenditures, compensation costs, diminished production, productivity, and reduced work participation^2^. This study aimed to validate an instrument for measuring the perception of occupational safety and health among Peruvian dentists.

## Methods

### Ethical considerations

The present study respected the bioethical principles of confidentiality, freedom, justice, respect and non-maleficence set out in the Declaration of Helsinki^18^. The study was approved by the ethics committee of the Faculty of Dentistry of the Universidad Nacional Federico Villarreal with opinion number 006-2024-COMITE-DE-ETICA dated 13 March 2024. In addition, participants gave their voluntary informed consent on the first page of the questionnaire.

### Study design

An analytical, prospective, observational, cross-sectional, analytical study with instrumental design was conducted. The manuscript was written according to the guidelines of strengthening the reporting of observational studies in epidemiology (STROBE)^19^.

### Sample size and participant selection

The study was conducted in the Peruvian capital between July and November 2024. The population consisted of 24,856 dentists in the Peruvian capital. The minimum sample size was calculated on the basis of Lloret-Segura et al.^20^, who recommended a minimum sample size of 200 cases, even under optimal conditions of high communalities and well-determined factors to perform exploratory factor analysis. Therefore, in Epidat 4.2 (*N* = 24,856), a formula for estimating a proportion with a finite population was taken into account, considering a significance level (α) = 0.05, a precision error of 5% and *p* = 0.5; therefore, we worked with a sample size of 379 participants (*n* = 379). Purposive sampling was used, which facilitated the selection of participants and allowed for more agile and efficient data collection.

Inclusion criteria.

- Dentists who voluntarily give their informed consent.
- Dentists affiliated to the Lima Dental Association.
- General and specialist dentists.
- Dentists who work in at least one establishment and report to a chief.

Exclusion criteria.

- Dentists who did not complete the questionnaire.

### Instrument preparation

The instrument for measuring the perception of occupational safety and health in dentists was adapted from Nogareda’s NTP 182 (Self-assessment survey of working conditions)^21^. This questionnaire in its original form was divided into 8 dimensions: D1 (Safety conditions), D2 (Environmental pollutants), D3 (Working environment), D4 (Job requirements), D5 (Work organization), D6 (Organization of prevention). D7 (Personal protection) and D8 (Warning symptoms) with a total of 188 items with Yes / No / Don’t know. Scoring was 1 point (correct) and 0 (incorrect). Sociodemographic characteristics of the dentists (age, gender, origin, marital status, academic degree and years of professional experience) were also included in the questionnaire.

### Procedure

The content of the questionnaire was reviewed and adapted by three experts in the field of dental research and validated by five experts with more than 15 years of professional experience (two researchers with a doctoral degree in public health, one researcher with a doctoral degree in education, one statistician, and one master’s degree in dentistry). Expert judgement carried out the validation in two stages. In the first stage, the experts indicated that the instrument could be applicable once the relevant corrections had been made to the observations made; after these observations were made, a first version of the instrument was obtained, divided into 7 dimensions: D1 (Safety conditions), D2 (Environmental conditions), D3 (Job requirements), D4 (Work organization), D5 (Prevention and health), D6 (Personal protection), and D7 (Warning symptoms), with a total of 114 items [see supplementary material]. Subsequently, in the second stage, the experts, in accordance with the Cosmin Guide^22^, carried out a validation for each item considering the criteria of relevance, comprehensiveness, and comprehensibility.

The questionnaire on the perception of occupational safety and health in dentists was transferred to Google Form^®^ and distributed using the self-administered survey technique by means of a link via social networks, WhatsApp^®^ and e-mails of the registered dentists in the Peruvian capital. Participants were automatically directed to the objective of the research and to the informed consent page by clicking on the link. Once they accepted, they were directed to the questionnaire with the instructions for completing it. Participants were free to opt out of the study at any point. Personal data such as name, telephone number, and address were not requested. The study was designed to be a one-time survey. Data were collected and stored in a Microsoft^®^ Excel 2019 spreadsheet and stored in a password-protected digital folder to which only the principal investigator had access. To avoid duplication of participation, participants were asked to initial their name along with their age (e.g., MILC42). The statistical package SPSS v.24.0 and the software Factor Analysis were used for data analysis.

### Statistical analysis

In order to ascertain content validity, the items were subjected to evaluation by five expert judges. The scores thus obtained were then used to calculate Aiken’s V coefficient, together with its 95% confidence interval, in accordance with the criteria of relevance, comprehensiveness, and comprehensibility. In this way, the critical point of Aiken’s V = 0.5 was taken into consideration^22^.

In order to validate the construct, a descriptive analysis was conducted in order to calculate the mean, variance, skewness, and kurtosis of the questionnaire items. The value ± 1.5 was considered for skewness and kurtosis. In addition, item-total response was assessed using tetrachoric correlation, as the responses to the questions were dichotomous^23^. Subsequently, an EFA was performed on the instrument, with a Kaiser-Meyer-Olkin measure of adequacy (KMO > 0.5) and Bartlett’s sphericity (*p* < 0.05) being considered acceptable. The number of dimensions of the questionnaire was determined according to principal component analysis in order to group and reduce the items^24,25^, after verification of the multivariate normality assumption (Mardia kurtosis).

CFA was then carried out, with parallel analysis of the variance explained by the items and the goodness of fit indices, e.g. χ^2^ (adjusted robust chi-square), WRMR, CFI, TLI, and RMSEA. The reliability of the questionnaire as a whole and of each of its dimensions was then analysed using Cronbach’s alpha.

## Results

Of the total number of participants, 52.5% were women, and 78.4% were originally from the Peruvian capital. In addition, the majority, 59.1%, were single. On the other hand, 52% were professional dentists with only a bachelor’s degree. Finally, the mean years of experience was 14.4 ± 13.2 years, and the mean age was 41.5 ± 14.5 years (Table 1).

**Table 1:** Sociodemographic characteristics of dentists.

Variable	Categories	Frequency	Percentage
Gender	Female	199	52.5
	Male	180	47.5
Origin	Capital	297	78.4
	Province	82	21.6
Marital status	Single	224	59.1
	Married or cohabiting	128	33.8
	Divorced or separated	27	7.1
Academic degree	Bachelor	197	52.0
	Master	140	36.9
	Doctor	42	11.1

	Mean	Median	SD
Years of professional experience	14.4	9	13.2
Age	41.5	38	14.5

*SD* standard deviation.

For the evaluation of the 114 items, the critical point of Aiken’s V (V) = 0.5 was considered. To eliminate an item, it was taken into account that the confidence interval does not contain such a critical value^26^. Therefore, after the evaluation of the five experiential judges, no item was removed, as the confidence interval did not pass the critical value according to the criteria of relevance, comprehensiveness, and comprehensibility^22^ (Table 2).

**Table 2:** Content validity of the relevance, comprehensiveness and comprehensibility of Q-OSH items.

Item	Relevance		Comprehensiveness		Comprehensibility	
	V	95% CI	V	95% CI	V	95% CI
1	0.93	0.90–0.95	0.94	0.92–0.93	0.93	0.90–0.95
2	0.95	0.93–0.97	0.95	0.93–0.97	0.94	0.92–0.93
3	0.94	0.92–0.93	0.95	0.93–0.97	0.94	0.92–0.93
4	0.94	0.92–0.93	0.95	0.93–0.97	0.94	0.92–0.93
5	0.95	0.93–0.97	0.95	0.93–0.97	0.93	0.90–0.95
6	0.94	0.92–0.93	0.95	0.93–0.97	0.95	0.93–0.97
7	0.95	0.93–0.97	0.95	0.93–0.97	0.95	0.93–0.97
8	0.95	0.93–0.97	0.95	0.93–0.97	0.95	0.93–0.97
9	0.95	0.93–0.97	0.95	0.93–0.97	0.95	0.93–0.97
10	0.95	0.93–0.97	0.94	0.92–0.93	0.94	0.92–0.93
11	0.95	0.93–0.97	0.94	0.92–0.93	0.95	0.93–0.97
12	0.95	0.93–0.97	0.93	0.90–0.95	0.94	0.92–0.93
13	0.94	0.92–0.93	0.93	0.90–0.95	0.95	0.93–0.97
14	0.94	0.92–0.93	0.94	0.92–0.93	0.92	0.89–0.94
15	0.95	0.93–0.97	0.95	0.93–0.97	0.95	0.93–0.97
16	0.94	0.92–0.93	0.95	0.93–0.97	0.94	0.92–0.93
17	0.95	0.93–0.97	0.95	0.93–0.97	0.95	0.93–0.97
18	0.95	0.93–0.97	0.95	0.93–0.97	0.94	0.92–0.93
19	0.95	0.93–0.97	0.95	0.93–0.97	0.95	0.93–0.97
20	0.94	0.92–0.93	0.94	0.92–0.93	0.94	0.92–0.93
21	0.95	0.93–0.97	0.95	0.93–0.97	0.95	0.93–0.97
22	0.94	0.92–0.93	0.95	0.93–0.97	0.95	0.93–0.97
23	0.95	0.93–0.97	0.95	0.93–0.97	0.94	0.92–0.93
24	0.95	0.93–0.97	0.95	0.93–0.97	0.94	0.92–0.93
25	0.95	0.93–0.97	0.95	0.93–0.97	0.94	0.92–0.93
26	0.95	0.93–0.97	0.95	0.93–0.97	0.95	0.93–0.97
27	0.95	0.93–0.97	0.94	0.92–0.93	0.94	0.92–0.93
28	0.93	0.90–0.95	0.94	0.92–0.93	0.94	0.92–0.93
29	0.95	0.93–0.97	0.94	0.92–0.93	0.94	0.92–0.93
30	0.95	0.93–0.97	0.95	0.93–0.97	0.95	0.93–0.97
31	0.94	0.92–0.93	0.94	0.92–0.93	0.94	0.92–0.93
32	0.95	0.93–0.97	0.95	0.93–0.97	0.95	0.93–0.97
33	0.95	0.93–0.97	0.95	0.93–0.97	0.95	0.93–0.97
34	0.95	0.93–0.97	0.95	0.93–0.97	0.95	0.93–0.97
35	0.95	0.93–0.97	0.95	0.93–0.97	0.95	0.93–0.97
36	0.94	0.92–0.93	0.95	0.93–0.97	0.95	0.93–0.97
37	0.95	0.93–0.97	0.95	0.93–0.97	0.95	0.93–0.97
38	0.94	0.92–0.93	0.95	0.93–0.97	0.95	0.93–0.97
39	0.95	0.93–0.97	0.94	0.92–0.93	0.94	0.92–0.93
40	0.94	0.92–0.93	0.94	0.92–0.93	0.94	0.92–0.93
41	0.95	0.93–0.97	0.94	0.92–0.93	0.94	0.92–0.93
42	0.95	0.93–0.97	0.95	0.93–0.97	0.95	0.93–0.97
43	0.95	0.93–0.97	0.95	0.93–0.97	0.95	0.93–0.97
44	0.95	0.93–0.97	0.95	0.93–0.97	0.95	0.93–0.97
45	0.95	0.93–0.97	0.95	0.93–0.97	0.95	0.93–0.97
46	0.94	0.92–0.93	0.95	0.93–0.97	0.95	0.93–0.97
47	0.95	0.93–0.97	0.94	0.92–0.93	0.94	0.92–0.93
48	0.95	0.93–0.97	0.95	0.93–0.97	0.94	0.92–0.93
49	0.95	0.93–0.97	0.95	0.93–0.97	0.94	0.92–0.93
50	0.94	0.92–0.93	0.95	0.93–0.97	0.95	0.93–0.97
51	0.94	0.92–0.93	0.95	0.93–0.97	0.95	0.93–0.97
52	0.95	0.93–0.97	0.95	0.93–0.97	0.95	0.93–0.97
53	0.95	0.93–0.97	0.95	0.93–0.97	0.95	0.93–0.97
54	0.95	0.93–0.97	0.94	0.92–0.93	0.94	0.92–0.93
55	0.94	0.92–0.93	0.94	0.92–0.93	0.94	0.92–0.93
56	0.95	0.93–0.97	0.95	0.93–0.97	0.95	0.93–0.97
57	0.95	0.93–0.97	0.95	0.93–0.97	0.95	0.93–0.97
58	0.95	0.93–0.97	0.95	0.93–0.97	0.95	0.93–0.97
59	0.95	0.93–0.97	0.95	0.93–0.97	0.95	0.93–0.97
60	0.95	0.93–0.97	0.95	0.93–0.97	0.95	0.93–0.97
61	0.94	0.92–0.93	0.94	0.92–0.93	0.94	0.92–0.93
62	0.94	0.92–0.93	0.94	0.92–0.93	0.94	0.92–0.93
63	0.95	0.93–0.97	0.94	0.92–0.93	0.94	0.92–0.93
64	0.94	0.92–0.93	0.94	0.92–0.93	0.94	0.92–0.93
65	0.95	0.93–0.97	0.95	0.93–0.97	0.94	0.92–0.93
66	0.94	0.92–0.93	0.95	0.93–0.97	0.95	0.93–0.97
67	0.95	0.93–0.97	0.95	0.93–0.97	0.95	0.93–0.97
68	0.95	0.93–0.97	0.95	0.93–0.97	0.95	0.93–0.97
69	0.95	0.93–0.97	0.94	0.92–0.93	0.95	0.93–0.97
70	0.95	0.93–0.97	0.95	0.93–0.97	0.95	0.93–0.97
71	0.95	0.93–0.97	0.95	0.93–0.97	0.95	0.93–0.97
72	0.95	0.93–0.97	0.94	0.92–0.93	0.94	0.92–0.93
73	0.95	0.93–0.97	0.95	0.93–0.97	0.95	0.93–0.97
74	0.95	0.93–0.97	0.95	0.93–0.97	0.95	0.93–0.97
75	0.95	0.93–0.97	0.94	0.92–0.93	0.94	0.92–0.93
76	0.95	0.93–0.97	0.94	0.92–0.93	0.94	0.92–0.93
77	0.95	0.93–0.97	0.94	0.92–0.93	0.94	0.92–0.93
78	0.95	0.93–0.97	0.94	0.92–0.93	0.94	0.92–0.93
79	0.95	0.93–0.97	0.94	0.92–0.93	0.95	0.93–0.97
80	0.95	0.93–0.97	0.94	0.92–0.93	0.94	0.92–0.93
81	0.94	0.92–0.93	0.95	0.93–0.97	0.95	0.93–0.97
82	0.95	0.93–0.97	0.95	0.93–0.97	0.95	0.93–0.97
83	0.95	0.93–0.97	0.95	0.93–0.97	0.95	0.93–0.97
84	0.94	0.92–0.93	0.95	0.93–0.97	0.95	0.93–0.97
85	0.95	0.93–0.97	0.95	0.93–0.97	0.94	0.92–0.93
86	0.95	0.93–0.97	0.95	0.93–0.97	0.95	0.93–0.97
87	0.94	0.92–0.93	0.95	0.93–0.97	0.95	0.93–0.97
88	0.95	0.93–0.97	0.95	0.93–0.97	0.95	0.93–0.97
89	0.95	0.93–0.97	0.94	0.92–0.93	0.94	0.92–0.93
90	0.95	0.93–0.97	0.94	0.92–0.93	0.94	0.92–0.93
91	0.95	0.93–0.97	0.94	0.92–0.93	0.94	0.92–0.93
92	0.95	0.93–0.97	0.94	0.92–0.93	0.94	0.92–0.93
93	0.95	0.93–0.97	0.95	0.93–0.97	0.95	0.93–0.97
94	0.95	0.93–0.97	0.95	0.93–0.97	0.94	0.92–0.93
95	0.94	0.92–0.93	0.95	0.93–0.97	0.95	0.93–0.97
96	0.95	0.93–0.97	0.95	0.93–0.97	0.95	0.93–0.97
97	0.95	0.93–0.97	0.95	0.93–0.97	0.94	0.92–0.93
98	0.94	0.92–0.93	0.95	0.93–0.97	0.94	0.92–0.93
99	0.95	0.93–0.97	0.95	0.93–0.97	0.94	0.92–0.93
100	0.95	0.93–0.97	0.94	0.92–0.93	0.95	0.93–0.97
101	0.95	0.93–0.97	0.94	0.92–0.93	0.95	0.93–0.97
102	0.94	0.92–0.93	0.94	0.92–0.93	0.95	0.93–0.97
103	0.95	0.93–0.97	0.94	0.92–0.93	0.95	0.93–0.97
104	0.95	0.93–0.97	0.94	0.92–0.93	0.95	0.93–0.97
105	0.94	0.92–0.93	0.95	0.93–0.97	0.95	0.93–0.97
106	0.94	0.92–0.93	0.95	0.93–0.97	0.95	0.93–0.97
107	0.94	0.92–0.93	0.93	0.90–0.95	0.95	0.93–0.97
108	0.94	0.92–0.93	0.95	0.93–0.97	0.94	0.92–0.93
109	0.95	0.93–0.97	0.95	0.93–0.97	0.95	0.93–0.97
110	0.95	0.93–0.97	0.94	0.92–0.93	0.94	0.92–0.93
111	0.95	0.93–0.97	0.94	0.92–0.93	0.93	0.90–0.95
112	0.95	0.93–0.97	0.94	0.92–0.93	0.94	0.92–0.93
113	0.94	0.92–0.93	0.94	0.92–0.93	0.93	0.90–0.95
114	0.95	0.93–0.97	0.94	0.92–0.93	0.95	0.93–0.97

*V* Aiken statistic, *95% CI* 95% confidence interval.

For the EFA, the skewness and kurtosis of the 114 items were calculated so that items with skewness and kurtosis > 1.5 had to be eliminated^27^. According to the excess of the skewness range, items 4, 6, 8, 10, 11, 11, 14, 18, 18, 25, 25, 30, 30, 31, 33, 35, 36, 38, 39, 40, 41, 42, 46, 49, 50, 52, 55, 57, 59, 63, 68, 69, 70, 71, 76, 77, 81, 85, 99, 104, 105, 112 were eliminated. Then, according to the excess of the kurtosis range, items 3, 9, 20, 21, 22, 26, 27, 28, 44, 54, 60, 65, 72, 80, 82, 83, 86, 89, 90, 92, 93, 94, 95, 96, 98, 100, 101, 102, 103, 107, 1113, 114 were eliminated (Table 3).

**Table 3:** Descriptive analysis, skewness and kurtosis of the 114 Q-OSH items.

Items	Mean	95% CI		Variance	Skewness	Kurtosis
		LL	UL			
1	0.682	0.620	0.740	0.217	− 0.782	− 1.388
2	0.787	0.730	0.840	0.168	− 1.405	− 0.030
3**	0.608	0.540	0.670	0.238	− 0.443	− 1.801
4*	0.829	0.780	0.880	0.142	− 1.752	1.063
5	0.645	0.580	0.710	0.229	− 0.606	− 1.631
6*	0.887	0.850	0.930	0.100	− 2.449	3.983
7	0.711	0.650	0.770	0.206	− 0.931	− 1.133
8*	0.876	0.830	0.920	0.108	− 2.292	3.243
9**	0.629	0.570	0.690	0.233	− 0.535	− 1.712
10*	0.855	0.810	0.900	0.124	− 2.025	2.092
11*	0.808	0.760	0.860	0.155	− 1.567	0.452
12	0.797	0.740	0.850	0.162	− 1.483	0.198
13	0.761	0.700	0.820	0.182	− 1.224	− 0.503
14*	0.882	0.840	0.920	0.104	− 2.368	3.596
15	0.705	0.650	0.770	0.208	− 0.903	− 1.184
16	0.705	0.650	0.770	0.208	− 0.903	− 1.184
17	0.787	0.730	0.840	0.168	− 1.405	− 0.030
18*	0.834	0.790	0.880	0.138	− 1.802	1.242
19	0.732	0.670	0.790	0.196	− 1.048	− 0.902
20**	0.366	0.300	0.430	0.232	0.559	− 1.686
21**	0.847	0.800	0.890	0.129	− 1.937	1.744
22**	0.613	0.550	0.680	0.237	− 0.466	− 1.781
23*	0.824	0.770	0.870	0.145	− 1.703	0.896
24	0.787	0.730	0.840	0.168	− 1.405	− 0.030
25*	0.855	0.810	0.900	0.124	− 2.025	2.092
26**	0.539	0.470	0.610	0.248	− 0.159	− 1.972
27**	0.461	0.390	0.530	0.248	0.159	− 1.972
28**	0.621	0.560	0.680	0.235	− 0.500	− 1.748
29	0.703	0.640	0.760	0.209	− 0.889	− 1.209
30*	0.861	0.810	0.910	0.120	− 2.087	2.346
31*	0.068	0.040	0.100	0.064	3.428	9.722
32	0.747	0.690	0.800	0.189	− 1.142	− 0.698
33*	0.847	0.800	0.890	0.129	− 1.937	1.744
34	0.695	0.630	0.760	0.212	− 0.848	− 1.280
35*	0.824	0.770	0.870	0.145	− 1.703	0.896
36*	0.837	0.790	0.890	0.137	− 1.828	1.335
37	0.779	0.720	0.830	0.172	− 1.348	− 0.185
38*	0.834	0.790	0.880	0.138	− 1.802	1.242
39*	0.821	0.770	0.870	0.147	− 1.680	0.816
40*	0.884	0.840	0.930	0.102	− 2.408	3.785
41*	0.811	0.760	0.860	0.154	− 1.589	0.521
42*	0.874	0.830	0.920	0.110	− 2.256	3.077
43	0.274	0.220	0.330	0.199	1.018	− 0.964
44**	0.424	0.360	0.490	0.244	0.310	− 1.902
45	0.742	0.680	0.800	0.191	− 1.110	− 0.769
46*	0.842	0.790	0.890	0.133	− 1.881	1.533
47	0.776	0.720	0.830	0.174	− 1.330	− 0.234
48	0.718	0.660	0.780	0.202	− 0.974	− 1.052
49*	0.845	0.800	0.890	0.131	− 1.909	1.637
50*	0.824	0.770	0.870	0.145	− 1.703	0.896
51	0.758	0.700	0.810	0.183	− 1.207	− 0.544
52*	0.863	0.820	0.910	0.118	− 2.119	2.481
53	0.795	0.740	0.850	0.163	− 1.463	0.138
54**	0.603	0.540	0.670	0.239	− 0.421	− 1.821
55*	0.839	0.790	0.890	0.135	− 1.854	1.432
56	0.779	0.720	0.830	0.172	− 1.348	− 0.185
57*	0.850	0.800	0.900	0.127	− 1.966	1.856
58	0.721	0.660	0.780	0.201	− 0.988	− 1.023
59*	0.805	0.750	0.860	0.157	− 1.546	0.386
60**	0.658	0.600	0.720	0.225	− 0.667	− 1.553
61	0.679	0.620	0.740	0.218	− 0.769	− 1.408
62	0.771	0.720	0.830	0.177	− 1.294	− 0.328
63*	0.834	0.790	0.880	0.138	− 1.802	1.242
64	0.755	0.700	0.810	0.185	− 1.191	− 0.584
65**	0.455	0.390	0.520	0.248	0.180	− 1.965
66	0.711	0.650	0.770	0.206	− 0.931	− 1.133
67	0.747	0.690	0.800	0.189	− 1.142	− 0.698
68*	0.921	0.890	0.960	0.073	− 3.131	7.781
69*	0.832	0.780	0.880	0.140	− 1.777	1.151
70*	0.921	0.890	0.960	0.073	− 3.131	7.781
71*	0.858	0.810	0.900	0.122	− 2.055	2.216
72**	0.568	0.500	0.630	0.245	− 0.277	− 1.921
73	0.747	0.690	0.800	0.189	− 1.142	− 0.698
74	0.747	0.690	0.800	0.189	− 1.142	− 0.698
75	0.776	0.720	0.830	0.174	− 1.330	− 0.234
76*	0.871	0.830	0.920	0.112	− 2.220	2.919
77*	0.913	0.880	0.950	0.079	− 2.942	6.636
78	0.776	0.720	0.830	0.174	− 1.330	− 0.234
79	0.755	0.700	0.810	0.185	− 1.191	− 0.584
80**	0.661	0.600	0.720	0.224	− 0.680	− 1.536
81*	0.826	0.780	0.880	0.144	− 1.727	0.978
82**	0.487	0.420	0.550	0.250	0.053	− 1.995
83**	0.613	0.550	0.680	0.237	− 0.466	− 1.781
84	0.763	0.710	0.820	0.181	− 1.241	− 0.461
85*	0.953	0.920	0.980	0.045	− 4.273	16.211
86**	0.595	0.530	0.660	0.241	− 0.387	− 1.848
87	0.784	0.730	0.840	0.169	− 1.385	− 0.083
88	0.726	0.670	0.780	0.199	− 1.018	− 0.964
89**	0.539	0.470	0.610	0.248	− 0.159	− 1.972
90**	0.571	0.510	0.640	0.245	− 0.288	− 1.915
91	0.705	0.650	0.770	0.208	− 0.903	− 1.184
92**	0.511	0.440	0.580	0.250	− 0.042	− 1.996
93**	0.571	0.510	0.640	0.245	− 0.288	− 1.915
94**	0.542	0.480	0.610	0.248	− 0.169	− 1.969
95**	0.637	0.570	0.700	0.231	− 0.571	− 1.673
96**	0.661	0.600	0.720	0.224	− 0.680	− 1.536
97	0.684	0.620	0.750	0.216	− 0.795	− 1.367
98**	0.471	0.410	0.540	0.249	0.116	− 1.984
99*	0.939	0.910	0.970	0.057	− 3.696	11.625
100**	0.503	0.440	0.570	0.250	− 0.011	− 1.997
101**	0.347	0.280	0.410	0.227	0.643	− 1.585
102**	0.350	0.290	0.410	0.228	0.631	− 1.601
103**	0.468	0.400	0.530	0.249	0.127	− 1.981
104*	0.963	0.940	0.990	0.035	− 4.930	22.248
105*	0.832	0.780	0.880	0.140	− 1.777	1.151
106	0.668	0.610	0.730	0.222	− 0.717	− 1.484
107**	0.418	0.350	0.480	0.243	0.332	− 1.888
108	0.255	0.200	0.310	0.190	1.126	− 0.734
109	0.255	0.200	0.310	0.190	1.126	− 0.734
110	0.276	0.220	0.340	0.200	1.003	− 0.994
111	0.305	0.240	0.370	0.212	0.848	− 1.280
112*	0.116	0.070	0.160	0.102	2.408	3.785
113**	0.624	0.560	0.690	0.235	− 0.512	− 1.736
114**	0.447	0.380	0.510	0.247	0.212	− 1.952

*Items removed according to the exceeded range for skewness. **Items eliminated according to the exceeded range for kurtosis.

After eliminating items according to excess skewness and kurtosis, 38 items remained. On the other hand, it was verified that the items did not meet the requirement of multivariate normality according to Mardia’s kurtosis = 1450.72 (*p* < 0.001), so instead of using Pearson’s correlation for the item-total correlation^28^, it was decided to use the tetrachoric correlation. Furthermore, this correlation is appropriate when item responses are dichotomous^23^. Then, according to the item-total tetrachoric correlation, those values that were < 0.30^20^ were eliminated, so items 108, 109, 110, and 111 were removed, leaving 34 items. Next, the communality of each remaining item was calculated, so it was decided to remove items 29, 43, 78, and 88, since they presented values lower than the minimum required (h^2^ = 0.30)^20^, leaving the questionnaire with 30 items (Table 4).

**Table 4:** Item-total correlation and communalities for each item.

Item	Correlation	Communalities
2	0.612	0.452
7	0.522	0.502
12	0.669	0.481
13	0.551	0.525
15	0.682	0.473
16	0.646	0.471
17	0.586	0.345
19	0.553	0.307
24	0.586	0.316
29**	0.740	0.293
32	0.796	0.425
34	0.768	0.360
37	0.856	0.413
43**	0.691	0.279
45	0.614	0.465
47	0.464	0.520
48	0.503	0.546
51	0.760	0.491
53	0.784	0.416
56	0.753	0.544
58	0.576	0.369
61	0.608	0.306
62	0.717	0.473
64	0.586	0.329
66	0.617	0.330
75	0.732	0.330
78**	0.561	0.247
79	0.586	0.344
84	0.637	0.310
87	0.824	0.337
88**	0.645	0.247
91	0.572	0.459
97	0.597	0.495
106	0.509	0.348
108*	0.230	
109*	0.273	
110*	0.243	
111*	0.167	

*Item to remove based on tetrachoric correlation (< 0.3). **Item to remove based on low communality (< 0.3).

The internal structure validity test yielded a KMO measure of 0.829, which was considered to be satisfactory. Furthermore, the Bartlett’s test of sphericity was found to be significant (*p* < 0.001) for the questionnaire comprising all 30 items. Conversely, the parallel analysis based on principal components indicated the extraction of four factors that explained 42.3% of the variance. However, given that the multivariate normality of kurtosis was not met, and in order to group and reduce the number of items^24,25^, the factor extraction method PCA was chosen. This resulted in the elimination of factor loadings lower than 0.4 (items 19, 34, 75, 84, and 87), leaving 25 items. Furthermore, as the correlations between factors were found to be low (< 0.4), it can be deduced that there was no multicollinearity^29,30^, thereby ensuring that the dimensions formed do not depend on a higher factor (Table 5).

**Table 5:** Principal component analysis and correlation between factors.

Initial item	F1	F2	F3	F4	Final item
45	0.658				1
47	0.595				2
48	0.694				3
58	0.559				4
61	0.539				5
62	0.683				6
64	0.540				7
66	0.557				8
2		0.537			9
7		0.525			10
17		0.481			11
24		0.503			12
37		0.579			13
51		0.668			14
53		0.590			15
56		0.692			16
12			0.593		17
13			0.749		18
15			0.642		19
16			0.570		20
79			0.433		21
32				0.449	22
91				0.671	23
97				0.654	24
106				0.537	25
Component	F1	F2	F3	F4	
F1	1.000				
F2	0.369	1.000			
F3	0.181	0.256	1.000		
F4	0.144	− 0.024	0.211	1.000	

*F* factor.

For the final 25-item questionnaire [see supplementary material], the parallel analysis was re-run and it was confirmed that it would be appropriate to consider four factors explaining 45.4% of the variance (Table 6).

**Table 6:** Explained variance in eigenvalues and parallel item analysis.

Variable	Explained variance in eigenvalues			Parallel analysis		
	Eigenvalues	Variance ratio	Cumulative variance ratio	Real data	Random mean	95th percentile of randomness
1	5.516	22.063	22.063	5.516*	1.500	1.577
2	2.453	9.813	31.876	2.453*	1.424	1.481
3	1.929	7.716	39.592	1.929*	1.363	1.410
4	1.463	5.851	45.443	1.463*	1.310	1.356
5	1.200	4.802	50.244	1.200	1.264	1.303
6	1.136	4.545	54.789	1.136	1.223	1.256
7	1.004	4.017	58.806	1.004	1.184	1.215
8	0.947	3.787	62.593	0.947	1.146	1.179
9	0.888	3.551	66.144	0.888	1.110	1.140
10	0.794	3.176	69.320	0.794	1.076	1.103
11	0.753	3.010	72.330	0.753	1.044	1.071
12	0.715	2.859	75.189	0.715	1.013	1.041
13	0.692	2.770	77.959	0.692	0.981	1.010
14	0.649	2.594	80.553	0.649	0.950	0.975
15	0.595	2.380	82.933	0.595	0.920	0.947
16	0.561	2.243	85.176	0.561	0.889	0.919
17	0.546	2.184	87.360	0.546	0.860	0.886
18	0.499	1.997	89.357	0.499	0.830	0.857
19	0.490	1.962	91.319	0.490	0.799	0.828
20	0.459	1.835	93.154	0.459	0.768	0.795
21	0.417	1.666	94.820	0.417	0.738	0.765
22	0.360	1.439	96.259	0.360	0.706	0.734
23	0.333	1.333	97.592	0.333	0.673	0.702
24	0.316	1.262	98.854	0.316	0.637	0.669
25	0.286	1.146	100.000	0.286	0.591	0.629

*Parallel analysis (PA) based on principal components, which recommends forming 4 dimensions.

The CFA showed adequate fit indices: Chi-square (χ^2^) = 321.071, df = 206, χ^2^/df = 1.559 (*p* < 0.001), SRMR = 0.047 (acceptable < 0. 08), CFI = 0.974 (good > 0.9), TLI = 0.963 (good > 0.9), WRMR = 0.045 (good fit < 1.0) and RMSEA = 0.038 (90% CI = 0.010–0.050)^31^.

Regarding the reliability of the overall instrument, Cronbach’s α was 0.846 (excellent), and according to its four dimensions, Cronbach’s α was 0.797, 0.773, 0.666, and 0.628, respectively, being these values acceptable.

## Discussion

The current literature identifies several instruments developed for evaluating occupational health and safety in dentists^14–17^. However, it is important to note that one of the instruments is interdisciplinary and not exclusively designed for dentists^14^, another does not address profession-specific risks or health-related aspects^15^, another assesses knowledge rather than the perception of risks and preventive measures^16^, and the last one omits important aspects, such as the physical conditions of the environment and work demands^17,32^. Consequently, this study aimed to validate an instrument for measuring the perception of occupational safety and health among Peruvian dentists.

The original instrument comprised 188 items, which were divided into eight dimensions^21^. Subsequently, an item-by‐item validation was undertaken in accordance with the COSMIN guidelines^22^, with particular attention given to the relevance, comprehensiveness, and comprehensibility of the 114 items. However, following a thorough evaluation, it was determined that no items required removal. Whilst these findings reinforced the content validity of the instrument and ensured that each item contributed significantly to the intended measurement, it is important to note that the Aiken V focuses solely on content validity; consequently, other forms of validation are essential for a comprehensive evaluation of the instrument^26^.

In accordance with methodological recommendations to prevent such extreme values from distorting the interpretation of the underlying factor structure, 76 items with skewness and kurtosis values higher than 1.5 were eliminated during the exploratory factor analysis^33–35^. High kurtosis is indicative of heavy tails in the data distribution, which can result in over or underestimation of factor loadings by assigning excessive weight to extreme values, thereby compromising the stability and validity of the factor model^34^. Similarly, high skewness indicates a substantial deviation from normality which complicates the accurate extraction of factors and the representation of latent dimensions^35^. Consequently, it was essential to remove these items to ensure that the analysis accurately reflects the underlying construct and to guarantee the integrity and robustness of the measurement instrument. Furthermore, such deviations from normality may lead to either an underestimation or an overestimation of factor loadings, making it challenging to accurately identify the latent dimensions. Accordingly, we employed analytical methods designed specifically for categorical items such as tetrachoric correlations, which align with the inherent distribution of dichotomous items and facilitate a more precise interpretation of the model^36^. Therefore, for the item total correlation, we opted to use tetrachoric correlations^23^ instead of Pearson’s correlation, which is appropriate for normally distributed items, eliminating items with values below 0.30 and retaining a total of 34 items. This approach minimizes distortions and ensures a more robust and reliable assessment of the questionnaire’s internal structure and psychometric validity^23^. Furthermore, the communality of each remaining item was calculated, and it was decided to remove 4 items as they had scores lower than the minimum required, indicating that the items did not contribute adequately to the construct being measured, so that only items that provided relevant information were retained^20,37^.

The KMO measure was adequate enough for the internal structure validity test. This means that the data can be used for factor analysis and that the questionnaire items are significantly linked to each other. It also found the Bartlett’s test of sphericity significant for the questionnaire containing all 30 items. This indicated that the item correlations are significantly different from zero and that a latent structure can be explored. This supports the suitability of the questionnaire to measure the proposed theoretical construct^20,38^. Moreover, the parallel analysis of the items indicated that it would be expedient to extract four factors that explained 42.3% of the variance, signifying that the four identified factors represent the most salient underlying dimensions in the data, thereby providing a simplified and understandable structure for further interpretation and analysis^20,38^. However, the multivariate normality of kurtosis wasn’t met, so PCA was used. Items with factor loadings of less than 0.4 were thrown out, leaving only 25 items that were representative. The loadings indicated that the items exhibited a weak relationship with the extracted factors, suggesting that they did not contribute significantly to the representation of the theoretical construct. Meanwhile, low inter-factor correlations (< 0.4) indicated the absence of multicollinearity, suggesting that the dimensions formed were independent and reflected distinct constructs without relying on a common underlying factor^29,39^. The final 25-item questionnaire was subjected to parallel principal component analysis, which confirmed that it would be appropriate to consider four factors explaining 45.4% of the variance. This finding suggests that a substantial proportion of the variability in responses can be attributed to these four factors, thereby validating their relevance in measuring the proposed theoretical construct^40^.

The CFA demonstrated adequate fit indices, thereby indicating that the proposed model reasonably fits the observed data and possesses sufficient flexibility to accommodate it without overfitting. This is imperative to ensure the validity and reliability of the inferences and conclusions derived from the analysis^41^. Furthermore, we observed an acceptable SRMR, which indicates minimal discrepancies between the observed covariances and the model predictions. Additionally, we identified a favorable CFI, indicating a robust model fit compared to a null model, thus validating the model^41^. It was found that the TLI worked well, which means that the model can clearly show the variance and covariance of the data. This observation suggests that the instrument is reliable and valid for quantifying the variables of interest. Finally, a WRMR and RMSEA were found to be low. These results show a good fit, which means that the model is good enough to show how the population’s covariance structure works. This makes us more confident in the proposed model’s validity^41^.

Concerning the reliability of the overall instrument, Cronbach’s α was determined to be 0.846, classifying it as excellent. For its four dimensions, Cronbach’s α was recorded as 0.797, 0.773, 0.666, and 0.628, respectively, which are considered acceptable values. According to these results, the instrument’s internal consistency is good enough for each dimension to be used in research. The instrument as a whole is good for measuring the concept being studied and gathering data in different areas of professional dental practice^42^. Although the Cronbach’s alpha value was slightly lower than 0.7 in the third and fourth dimensions of the present instrument, this can be considered justifiable, given the complexity or multidimensionality of the construct assessed. In this sense, the interpretation of alpha should be framed within the theoretical and empirical context of the construct^43,44^. Furthermore, in dimensions with few dichotomous items, the alpha value may be lower, as dichotomous responses tend to have less variability, which slightly reduces the internal consistency of these dimensions. This is because dichotomous items limit variability compared to Likert-type items^44,45^.

The research’s most significant contribution is the simplification of a general instrument for safety at work in the dental field. The original NTP-182 quiz had 188 questions spread out over eight dimensions. The simplified version has just 25 questions spread out over four dimensions: F1 (work demands and well-being), F2 (ergonomics and physical conditions of the environment), F3 (safety and risk prevention), and F4 (working conditions and worker protection). The dimensions of this new version have been reformulated according to the content of the items and the literature related to dentistry, offering a simplified version of the instrument while preserving the validity of the questionnaire by including representative items, thus ensuring the relevance of the questions. In addition, it improved the psychometric properties by increasing reliability and validity after the removal of irrelevant items^46^. It also reduces the time needed and improves comprehension for the development and application of the questionnaire, thus facilitating its use in time-critical situations with a more agile interpretation of the results^47^. Dimension F1 (work demands and well-being) assesses how work demands impact employees’ health, including adequate sleep, recovery from fatigue, sufficient breaks, flexibility in work rhythm and schedule, as well as the possibility of short absences^48^. The F2 dimension (ergonomics and physical conditions of the environment) is concerned with the prevention of injuries through the adequate design of the workspace. This includes the adequate protection of cables and plugs, as well as the necessary safety measures for the use of electrical instruments. It also covers aspects such as the lighting and temperature of the workspace, the maintenance of clean and disinfected areas, and the availability of ergonomic seating that ensures sufficient space to vary the position of the legs and perform the work comfortably^49,50^. Dimension F3 (safety and risk prevention) emphasizes the establishment of protocols and safety measures that minimize occupational risks. Such measures include the evaluation of warning signs for hazards, the availability of fire-fighting equipment such as fire extinguishers and hoses, the existence of rules for the handling and transport of dental materials and supplies, and regular equipment checks and consultation with staff in occupational decisions^9,51^. Finally, dimension F4 (working conditions and worker protection) assesses a fair and safe working environment, which includes the existence of adequate spaces for handling chemical supplies, the availability of staff trained in first aid, and the presence of posters indicating the mandatory use of personal protective equipment (PPE)^52^. The present 25-item instrument has been designed to facilitate a rapid diagnosis of the occupational safety and health situation, thereby encouraging greater staff participation. The results obtained from this study will facilitate dentists’ understanding of the risks associated with their practice, thus fostering a culture of self-care and responsibility that will, in turn, improve their health and, consequently, the quality of service provided to patients^32^.

Among the limitations of the present study, a stability analysis of the instrument was not performed, as it was not possible to measure the precision and accuracy of the instrument over time in various contexts. In addition, the survey was conducted virtually, as the geographical extension of Metropolitan Lima made it difficult to access dentists in person according to their available schedules. It is imperative to acknowledge the inherent limitations of virtual administration, including reduced participation and the potential for item misinterpretation due to the absence of direct interaction. Consequently, subsequent pilot testing or cognitive interviews will be essential for identifying and resolving any potential issues with online administration. Another limitation was that the present study was conducted only in the Peruvian capital. Nevertheless, the study provides a basis for further research to validate or improve the applicability of the instrument in various contexts and geographic regions. It is important to recognise that the process of validating an instrument is a continuous one, as it is not feasible to assess all psychometric properties for every aspect of validity and reliability in all potential applications.

In view of the findings, it is recommended that future research should investigate convergent validity when comparing this instrument with others that measure analogous constructs, and discriminant validity when comparing this instrument with others that measure constructs unrelated to the construct of interest in this study. In addition, it is recommended that structural invariance between genders be assessed. Additionally, it is recommended to test and retest this questionnaire by altering the order of the questions on two separate occasions and to evaluate the concordance of the scores^53^. It is recommended that the scope of the questionnaire be expanded to include dimensions such as work-life balance, institutional policies, and socioeconomic influences, as these may indirectly affect perceptions of occupational safety and health, thereby enabling a more comprehensive evaluation of the construct. Therefore, it is recommended that qualitative methods (e.g. interviews, focus groups) be incorporated to complement and contextualize the quantitative findings. This broader approach would facilitate a more comprehensive evaluation of the underlying construct and potentially enhance the instrument’s explanatory power in terms of variance^54,55^. Finally, to effectively implement an occupational safety and health measurement instrument in dental practice and enhance workplace safety standards, it is recommended that professional associations incorporate it into routine safety audits and training programs. The data collected should be used to identify critical areas for improvement and to design interventions that address the identified risks. In addition, establishing a continuous monitoring system will facilitate tracking changes over time and adjusting safety protocols in response to new challenges. This approach will foster a culture of continuous improvement in occupational safety and health within dental practice.

## Conclusion

In conclusion, recognizing the limitations of the present study, the simplified questionnaire to assess dentists’ perceptions of occupational safety and health has been demonstrated to be both valid and reliable. Its utilization for research purposes is recommended, with a focus on the following four dimensions: work demands and well-being, ergonomics and physical conditions of the environment, safety and risk prevention, and working conditions and worker protection. To ensure the validity of the findings, it is advised that the questionnaire be administered to a larger sample in a range of social and geographical contexts.

## Electronic supplementary material

Below is the link to the electronic supplementary material.

Supplementary Material 1

## Data Availability

The datasets used and/or analyzed during the current study are available from the corresponding author on reasonable request.

## Abbreviations

CFA: Confirmatory factor analysis
CFI: Comparative fit index
CI: Confidence interval
df: Degrees of freedom
COSMIN: COnsensus-based Standards for Measuring INstruments
EFA: Exploratory factor analysis
ILO: International Labour Organization
IWHAI: Interdisciplinary Worker Health Approach Instrument
OSH: Occupational Safety and Health
PCA: Principal component analysis
SDG: Sustainable Development Goal
SPSS: Statistical Package for the Social Sciences
URSULA: Union of Latin American University Social Responsibility
USR: University Social Responsibility
WHO: World Health Organization
SRMR: Standardized root mean square residual
TLI: Tucker and Lewis index
WRMR: Weighted root mean square residual
RMSEA: Root mean square error of approximation
