# Supplementary material for: Validation of an instrument to measure the perception of occupational safety and health among Peruvian dentists
Source: Sci Rep. 2025 May 2;15:15357. doi: 10.1038/s41598-025-00395-7 (PMC12048651; doi:10.1038/s41598-025-00395-7)
Supplement: Supplementary file 1 — Supplementary Material 1 [file 41598_2025_395_MOESM1_ESM.docx]

**Supplementary Material:**

**Initial version of the questionnaire adapted and validated by experienced judges (114 items): Perceptions of occupational safety and health**

| **Item** |  | Yes | No | I don’t know |
| --- | --- | --- | --- | --- |
|  | **Safety Conditions** | | | |
|  | **Equipment** |  |  |  |
| 1 | Are the surfaces and furniture well-polished (with no sharp edges)? |  |  |  |
| 2 | Are the cables of dental equipment (dental unit, compressor, oven, X-ray equipment, etc.) and/or plugs properly protected? |  |  |  |
| 3 | Do the dental equipment have switches or other emergency stop systems in case of an emergency? |  |  |  |
|  | **Instruments** | | | |
| 4 | Are the instruments well-maintained? |  |  |  |
| 5 | Are the instruments with tips properly sharpened? |  |  |  |
| 6 | When not in use, are the instruments properly stored in their designated place? |  |  |  |
| 7 | If the instruments are electrical, do they have the proper protection? |  |  |  |
| 8 | Are appropriate instruments available for patient care in each case? |  |  |  |
|  | **Space** | | | |
| 9 | Does the distance between the dental equipment prevent their moving parts from hitting people or other equipment? |  |  |  |
| 10 | Do the cabinets or furniture allow for the organized storage of dental materials and supplies? |  |  |  |
| 11 | Are the floors clean and slip-resistant in your workplace? |  |  |  |
| 12 | Are there warning signs and/or hazard alerts in your workplace? |  |  |  |
| 13 | Does your workplace have fire-fighting equipment, such as a fire hose and extinguishers? |  |  |  |
| 14 | Are the emergency exits clearly marked? |  |  |  |
|  | **Handling and transportation** | | | |
| 15 | Are there regulations in your workplace regarding the handling and transportation of dental materials and supplies? |  |  |  |
| 16 | Are regular inspections and maintenance performed on the dental equipment? |  |  |  |
|  | **Environmental conditions** | | | |
|  | **Lighting** | | | |
| 17 | Is the lighting positioned in a way that prevents glare and reflections? |  |  |  |
| 18 | Are the light bulbs and windows kept clean? |  |  |  |
| 19 | Are burnt-out light bulbs replaced promptly? |  |  |  |
| 20 | Are light level measurements conducted in your workplace? |  |  |  |
| 21 | Do the different areas of your workplace have the minimum lighting levels required to provide proper patient care? |  |  |  |
|  | **Temperature** | | | |
| 22 | Are heat sources properly insulated? |  |  |  |
| 23 | Does your workplace have general ventilation? |  |  |  |
| 24 | Do you consider the temperature in your workplace to be appropriate? |  |  |  |
| 25 | Is the clothing you're allowed to wear appropriate for the amount of working hours and environmental temperature? |  |  |  |
| 26 | Is maintenance performed on the ventilation systems in your workplace? |  |  |  |
|  | **Vibrations** | | | |
| 27 | Are the dental equipment and/or instruments that produce vibrations equipped with damping systems? |  |  |  |
| 28 | Are the dental equipment that produce vibrations properly isolated? |  |  |  |
|  | **Radiation** | | | |
| 29 | Is the area where the X-ray equipment is located clearly marked? |  |  |  |
| 30 | Regarding the use of X-rays, have you been informed of the risks involved and the preventive measures that should be considered? |  |  |  |
| 31 | In your workplace, do they have dosimeters to measure the amount of radiation? |  |  |  |
|  | **Chemical and/or biological contaminants** | | | |
| 32 | Are there proper environments for handling dental chemical supplies (workplace)? |  |  |  |
| 33 | Are the chemical products and supplies clearly labeled (workplace)? |  |  |  |
| 34 | Are there suitable environments for handling dental chemical supplies (workplace)? |  |  |  |
| 35 | Are there suitable environments for cleaning dental instruments (workplace)? |  |  |  |
| 36 | Are the cleaning areas and cafeterias separated from the work area (workplace)? |  |  |  |
| 37 | Are the work areas, changing rooms, restrooms, and cafeterias kept in perfect cleaning and disinfection conditions (workplace)? |  |  |  |
| 38 | In your workplace, are you always provided with supplies or materials for proper personal hygiene (e.g., for washing hands after each dental procedure, before eating, changing clothes when leaving work, etc.)? |  |  |  |
| 39 | Are there protocols for the handling of dental waste? |  |  |  |
|  | **Job requirements** | | | |
|  | **Physical fatigue** | | | |
| 40 | Is the physical effort required by your job appropriate for your capacity? |  |  |  |
| 41 | Is the physical effort required by your job appropriate for the temperature in your workplace? |  |  |  |
| 42 | Is the physical effort required by your job appropriate for your age? |  |  |  |
| 43 | In your workplace, has metabolic consumption been evaluated based on the activity you perform? |  |  |  |
| 44 | Are you required to take breaks for rest in your workplace? |  |  |  |
|  | **Mental load** | | | |
| 45 | From the perspective of nervous fatigue, do you consider your usual work pace to be appropriate? |  |  |  |
| 46 | From the perspective of nervous fatigue, do you believe the tasks required of you are within your capability? |  |  |  |
| 47 | Does your job allow you to sleep well at night? |  |  |  |
| 48 | Do you think the recovery from fatigue between one workday and the next is sufficient? |  |  |  |
| 49 | Is it easy to maintain concentration in your workplace? |  |  |  |
|  | **Ergonomics in the workplace** | | | |
| 50 | Can you work seated in different positions according to the clock hands, using the patient's mouth as the reference point? |  |  |  |
| 51 | Is your seat comfortable? |  |  |  |
| 52 | Is the work chair adjustable? |  |  |  |
| 53 | Do you have enough space to change the position of your legs and knees? |  |  |  |
| 54 | Can you rest your arms? |  |  |  |
| 55 | Is the height of the surface where you place the instruments comfortable for you? |  |  |  |
| 56 | In general, do you have enough space to perform your work comfortably? |  |  |  |
| 57 | At the end of the workday, would the fatigue you feel be considered normal? |  |  |  |
|  | **Work organization** | | | |
|  | **Workday** | | | |
| 58 | Are the number and total duration of breaks during the workday sufficient? |  |  |  |
| 59 | Does the workload allow you to organize and distribute tasks effectively in order to complete your work for the day? |  |  |  |
| 60 | Are you asked for your opinion regarding shift or schedule changes? |  |  |  |
| 61 | Can you choose your schedule? |  |  |  |
| 62 | Do you consider the work schedule distribution to be adequate? |  |  |  |
|  | **Pace** | | | |
| 63 | Do you think the time allocated to the task you perform is adequate? |  |  |  |
| 64 | Can you leave your work for a few minutes without needing a replacement? |  |  |  |
| 65 | Are there 'replacements' to substitute you when you cannot leave your position? (for a few minutes of absence) |  |  |  |
| 66 | Can you vary your work pace without disrupting production throughout the day? |  |  |  |
| 67 | Are you the one who sets the work pace? |  |  |  |
|  | **Automation** | | | |
| 68 | Does your work allow you to apply your skills and knowledge? |  |  |  |
| 69 | Can you organize your work your way? |  |  |  |
| 70 | Can you intervene in case of an error or incident to control and correct your work? |  |  |  |
| 71 | Do you consider your work to be varied? |  |  |  |
|  | **Communication and cooperation** | | | |
| 72 | Do you work with an assistant to perform dental procedures? |  |  |  |
| 73 | If you work individually, can you talk to other colleagues during work? |  |  |  |
| 74 | Are there colleagues less than 5 meters away from your work station? |  |  |  |
| 75 | Are workers trained when new equipment or work methods are introduced? |  |  |  |
| 76 | Do you have friends at work? |  |  |  |
| 77 | Can you speak your mind, as long as it's done with respect? |  |  |  |
| 78 | Do you know the ideas of other colleagues, e.g., religion, politics, or their expectations? |  |  |  |
|  | **Management style and participation** | | | |
| 79 | Does your immediate supervisor ask for your opinion on decisions that affect the work you do? |  |  |  |
| 80 | When your immediate supervisor assigns you a new task, does he/she discuss with you how to carry it out? |  |  |  |
| 81 | Can you give direct suggestions to your superiors? |  |  |  |
| 82 | Are there suggestion boxes at your workplace? |  |  |  |
| 83 | Do you think that the suggestions given by workers are taken into account? |  |  |  |
|  | **Status** | | | |
| 84 | Does your work enjoy prestige among your colleagues? |  |  |  |
| 85 | Do you think your work is important within the overall process? |  |  |  |
| 86 | Is your employment contract permanent? |  |  |  |
| 87 | Is the training of workers adequate for the tasks they perform? |  |  |  |
| 88 | In general, are you familiar with everything that is done at the institution where you work? |  |  |  |
| 89 | Do you have opportunities for promotion or advancement? |  |  |  |
| 90 | Compared to other positions, is your compensation appropriate for the demands of your role? |  |  |  |
|  | **Prevention and health** | | | |
|  | **Legislation** | | | |
| 91 | Based on the work you do; do you know what benefits you are entitled to? |  |  |  |
| 92 | Are there copies of the Occupational Health and Safety Law at your workplace? |  |  |  |
| 93 | Have you read the workplace regulations at your place of employment? |  |  |  |
|  | **Risk control** | | | |
| 94 | Is there a safety and health monitor or committee at your workplace that informs employees about workplace accidents? |  |  |  |
| 95 | Does your workplace have medical services |  |  |  |
| 96 | Is there a well-stocked first aid kit that is periodically checked at your workplace? |  |  |  |
| 97 | Are there people at your workplace trained in first aid to provide assistance? |  |  |  |
| 98 | Are periodic medical exams conducted for employees based on the risks they are exposed to, and are the results always communicated to them? |  |  |  |
| 99 | Are you aware of the potential occupational diseases you may be exposed to in your workplace? |  |  |  |
| 100 | If a workplace accident were to occur, is there any document or form to record what happened? |  |  |  |
| 101 | At your workplace, is a risk assessment conducted before starting work? |  |  |  |
| 102 | Are the levels of contamination in your workplace measured and controlled? |  |  |  |
| 103 | Does your workplace have effective advice, either internal or external, on accident prevention? |  |  |  |
|  | **Personal protection** | | | |
| 104 | Do you use personal protective equipment (PPE) such as hats, glasses, face shields, masks, lab coats, gloves, boots, among others? |  |  |  |
| 105 | Does your workplace provide you with PPE? |  |  |  |
| 106 | Are there posters at your workplace indicating the mandatory use of PPE? |  |  |  |
|  | **Alert symptoms** | | | |
| 107 | Due to your work, do you feel fatigued? |  |  |  |
| 108 | Due to your work, do you have trouble sleeping? |  |  |  |
| 109 | Due to your work, do you feel heaviness in your head or experience dizziness? |  |  |  |
| 110 | Due to your work, do you feel irritated? |  |  |  |
| 111 | Do you find it hard to concentrate on other activities because you're thinking about work-related matters? |  |  |  |
| 112 | Due to your work, do you take tranquilizers? |  |  |  |
| 113 | Due to your work, do you experience pain in your neck, back, or lower back? |  |  |  |
| 114 | Due to your work, do you experience eye discomfort (glare and/or blinking)? |  |  |  |

**Spanish version**

|  | Si | No | No se |
| --- | --- | --- | --- |
| **Condiciones de seguridad** | | | |
| **Equipos** |  |  |  |
| Los mobiliarios ¿Están bien pulimentados? (no tienen bordes cortantes) |  |  |  |
| ¿Los cables de los equipos odontológicos (unidad dental, compresora, estufa, equipo de Rx, etc) y/o enchufes están protegidos? |  |  |  |
| ¿Los equipos odontológicos cuentan con interruptores u otros sistemas de paro de alerta ante una emergencia? |  |  |  |
| **Instrumental** | | | |
| ¿Los instrumentos están bien conservados? |  |  |  |
| ¿Los instrumentos que poseen puntas están bien afilados? |  |  |  |
| Cuando los instrumentos no se utilizan, ¿Están bien guardados en el lugar que corresponde? |  |  |  |
| ¿Si el instrumental es eléctrico tienen la protección adecuada? |  |  |  |
| ¿Se dispone en cada caso de instrumentos adecuados para la atención de pacientes? |  |  |  |
| **Espacio** | | | |
| ¿La distancia entre los equipos odontológicos impide que sus elementos móviles golpeen a personas u otros equipos? |  |  |  |
| ¿Loa armarios o muebles permiten guardar los materiales e insumos odontológicos de manera ordenada? |  |  |  |
| ¿Están los pisos limpios y son antideslizantes en tu lugar de trabajo? |  |  |  |
| ¿Existen señales de atención y/o advertencias frente al peligro en tu lugar de trabajo? |  |  |  |
| ¿Cuenta tu lugar de trabajo con equipos contra incendios, por ejemplo, manguera para bomberos y extintores? |  |  |  |
| ¿Están señalizadas las salidas de emergencia? |  |  |  |
| **Manipulación y transporte** | | | |
| ¿Existen normas dictadas en tu lugar de trabajo respecto a la manipulación y transporte de materiales e insumos odontológicos? |  |  |  |
| ¿Se realizan revisiones y mantenimiento periódico de los equipos odontológicos? |  |  |  |
| **Condiciones medioambientales** | | | |
| **Iluminación** | | | |
| ¿La luz está situada de forma que impida deslumbramientos y reflejos? |  |  |  |
| ¿Se mantienen los focos de luz y ventanas limpias? |  |  |  |
| ¿Los focos fundidos son sustituidos rápidamente? |  |  |  |
| ¿Se realizan mediciones del nivel de luz en tu lugar de trabajo? |  |  |  |
| ¿Los distintos espacios de tu lugar de trabajo disponen de los niveles mínimos de iluminación para realizar una adecuada atención al paciente? |  |  |  |
| **Temperatura** | | | |
| ¿Los focos de calor están aislados convenientemente? |  |  |  |
| ¿Dispone su lugar de trabajo de ventilación general? |  |  |  |
| ¿Consideras que la temperatura de tu lugar trabajo es la adecuada? |  |  |  |
| ¿La ropa que te permiten utilizar es adecuada según la cantidad de tiempo de trabajo y temperatura ambiental? |  |  |  |
| ¿Se realiza un mantenimiento de los sistemas de ventilación en tu lugar de trabajo? |  |  |  |
| **Vibraciones** | | | |
| ¿Los equipos odontológicos y/o instrumental que producen vibraciones están dotados de sistemas de amortiguación? |  |  |  |
| ¿Los equipos odontológicos que producen vibraciones están aislados? |  |  |  |
| **Radiaciones** | | | |
| ¿Está señalizado el espacio donde se ubica el equipo de rayos X |  |  |  |
| Con respecto al uso de los rayos X ¿Te han informado de los riesgos a los que estás sometido y las medidas preventivas que se deben tener en cuenta? |  |  |  |
| En tu lugar de trabajo, ¿Disponen de dosímetros para medir la cantidad de radiación? |  |  |  |
| **Contaminantes químicos y/o biológicos** | | | |
| En tu lugar de trabajo ¿Existen protocolos para el manejo de productos químicos? |  |  |  |
| ¿Los productos e insumos químicos están claramente etiquetados? |  |  |  |
| ¿Existen ambientes adecuados para el manejo de insumos químicos odontológicos? |  |  |  |
| ¿Existen ambientes adecuados para el lavado del instrumental odontológico? |  |  |  |
| ¿Se encuentran las zonas de aseo y comedores, aislados de la zona de trabajo? |  |  |  |
| ¿Se mantienen las zonas de trabajo, vestuario, aseo y comedores en perfectas condiciones de limpieza y desinfección? |  |  |  |
| En tu lugar de trabajo, ¿Siempre te proveen de insumos o materiales para una buena higiene personal (por ejm, para lavarse las manos después de cada procedimiento odontológicos, antes de comer, cambiarse ropa al salir del trabajo, etc.)? |  |  |  |
| ¿Existen protocolos para el manejo de residuos odontológicos? |  |  |  |
| **Exigencias del puesto** | | | |
| **Fatiga física** | | | |
| El esfuerzo físico que te exige el trabajo ¿están adecuados para tu capacidad? |  |  |  |
| El esfuerzo físico que te exige el trabajo ¿están adecuados según la temperatura de tu lugar de trabajo? |  |  |  |
| El esfuerzo físico que te exige el trabajo ¿están adecuados para tu edad? |  |  |  |
| En tu lugar de trabajo ¿Han evaluado el consumo metabólico según la actividad que realizas? |  |  |  |
| ¿Te exigen hacer pausas a modo de descanso, en tu lugar de trabajo? |  |  |  |
| **Carga mental** | | | |
| Desde el punto de vista de la fatiga nerviosa ¿Consideras que tu ritmo habitual de trabajo es adecuado? |  |  |  |
| Desde el punto de vista de la fatiga nerviosa ¿Crees que la actividad que se te exige es la que tú puedes realizar? |  |  |  |
| ¿Tu trabajo te permite dormir bien por las noches? |  |  |  |
| ¿Crees que la recuperación de la fatiga entre una jornada de trabajo y la siguientes es suficiente? |  |  |  |
| ¿En tu lugar de trabajo es fácil mantener la concentración? |  |  |  |
| **Ergonomía en el trabajo** | | | |
| ¿Puedes trabajar sentado en diferentes posiciones según las manecillas del reloj, tomando como centro de referencia la boca del paciente? |  |  |  |
| ¿Tu asiento es cómodo? |  |  |  |
| ¿Es ajustable la silla de trabajo? |  |  |  |
| ¿Tienes espacio suficiente para variar la posición de piernas y rodillas? |  |  |  |
| ¿Puedes apoyar los brazos? |  |  |  |
| La altura de la superficie donde colocas el instrumental ¿te resulta cómoda? |  |  |  |
| En general ¿Dispones de espacio suficiente para realizar el trabajo con holgura? |  |  |  |
| Al finalizar la jornada laboral ¿El cansancio que sientes podría calificarse como normal? |  |  |  |
| **Organización del trabajo** | | | |
| **Jornada** | | | |
| ¿El número y la duración total de las pausas durante la jornada laboral, son suficientes? |  |  |  |
| ¿La carga laboral te permite organizarte y distribuir bien las tareas, a fín de cumplir con tu trabajo del día? |  |  |  |
| ¿Te piden opinión para el cambio de turno u horario? |  |  |  |
| ¿Puedes escoger tu horario? |  |  |  |
| ¿Consideras adecuada la distribución del horario de trabajo? |  |  |  |
| **Ritmo** | | | |
| ¿Consideras que el tiempo asignado a la tarea que realizas es el adecuado? |  |  |  |
| ¿Puedes abandonar tu trabajo por unos minutos sin necesidad de que te sustituyan? |  |  |  |
| ¿Existen “reemplazos” para sustituirte cuando no se puede abandonar el puesto? (para ausentarte unos minutos) |  |  |  |
| ¿Puedes variar tu ritmo de trabajo sin perturbar la producción a lo largo de la jornada? |  |  |  |
| ¿Eres tú el que marca el ritmo de trabajo? |  |  |  |
| **Automatización** | | | |
| ¿Tu trabajo te permite aplicar tus habilidades y conocimientos? |  |  |  |
| ¿Puedes organizar tu trabajo a tu manera? |  |  |  |
| ¿Puedes intervenir en caso de error o incidente para controlar y corregir tu trabajo? |  |  |  |
| ¿Consideras que tu trabajo es variado? |  |  |  |
| **Comunicación y cooperación** | | | |
| ¿Trabajas con un asistente para realizar procedimientos odontológicos? |  |  |  |
| ¿Si trabajas de forma individual, puedes hablar con otros compañeros durante el trabajo? |  |  |  |
| ¿Hay compañeros de trabajo a menos de 5 m. de tu puesto de trabajo? |  |  |  |
| ¿Los trabajadores son capacitados cuando se introducen nuevos equipos o métodos de trabajo? |  |  |  |
| ¿Tienes amigos en el trabajo? |  |  |  |
| ¿Se puede decir lo que uno piensa, siempre y cuando se haga con respeto? |  |  |  |
| ¿Conoces las ideas de otros compañeros, por ejm, religión, política, o sobre sus expectativas? |  |  |  |
| **Estilo de mando y participación** | | | |
| ¿Tu jefe inmediato te pide opinión en las decisiones que afectan el trabajo que realizas? |  |  |  |
| Cuando tu jefe inmediato te encarga una nueva tarea ¿Discute contigo la forma de llevarla a cabo? |  |  |  |
| ¿Puedes dar directamente sugerencias a tus superiores? |  |  |  |
| ¿Hay buzones de sugerencias en tu lugar de trabajo? |  |  |  |
| ¿Crees que se tienen en cuenta las sugerencias que dan los trabajadores? |  |  |  |
| **Status** | | | |
| ¿Goza tu trabajo de prestigio entre tus compañeros? |  |  |  |
| ¿Piensas que tu trabajo es importante dentro del proceso general? |  |  |  |
| ¿Tu contrato de trabajo es fijo? |  |  |  |
| La preparación de los trabajadores ¿Es adecuada al trabajo que realizan? |  |  |  |
| En general, ¿conoces todo lo que se hace en la institución donde trabajas? |  |  |  |
| ¿Tienes posibilidades de promoción o ascenso? |  |  |  |
| Comparado con otros puestos ¿Es adecuada tu remuneración según las exigencias del puesto que desempeñas? |  |  |  |
| **Prevención y salud** | | | |
| **Legislación** | | | |
| Según el trabajo que realizas ¿Sabes a qué prestaciones tienes derecho? |  |  |  |
| ¿En tu lugar de trabajo hay ejemplares de la Ley de Seguridad y Salud en el Trabajo? |  |  |  |
| ¿Has leído el reglamento de trabajo del lugar donde laboras? |  |  |  |
| **Control de riesgos** | | | |
| ¿Existe en tu lugar de trabajo, un vigilante o comité de seguridad y salud que informe a los trabajadores sobre accidentes laborales? |  |  |  |
| ¿Tu lugar de trabajo tiene servicio médico? |  |  |  |
| ¿Hay botiquín suficientemente dotado y revisado periódicamente en tu lugar de trabajo? |  |  |  |
| ¿Hay personas que puedan prestar los primeros auxilios con la formación de socorristas? |  |  |  |
| ¿Se realizan exámenes médicos periódicos a los trabajadores, en función de los riesgos a los que están expuestos y siempre es informado de los resultados? |  |  |  |
| ¿Eres consciente de las posibles enfermedades profesionales a la que te encuentras expuesto en tu lugar de trabajo? |  |  |  |
| Si ocurriera un accidente de trabajo ¿existe algún documento o ficha para registrar lo sucedido? |  |  |  |
| En tu lugar de trabajo ¿se hace un análisis de riesgos antes de comenzar a laborar? |  |  |  |
| ¿Se miden y controlan los niveles de contaminación existentes en tu lugar de trabajo? |  |  |  |
| Tu lugar de trabajo ¿Dispone de asesoramiento eficaz, ya sea propio o externo, en materia de prevención de accidentes? |  |  |  |
| **Protección personal** | | | |
| ¿Utilizas equipos de protección personal (EPP) como gorros, lentes, protector facial, mascarillas, mandil, guantes, botas, entre otros? |  |  |  |
| ¿Tu lugar de trabajo te proporciona EPP? |  |  |  |
| ¿En tu lugar de trabajo hay afiches que indiquen el uso obligatorio de EPP? |  |  |  |
| **Síntomas de alerta** | | | |
| A causa de tu trabajo ¿Te sientes fatigado/a? |  |  |  |
| A causa de tu trabajo ¿Te cuesta dormir? |  |  |  |
| A causa de tu trabajo ¿Tienes la cabeza pesada o sientes mareos? |  |  |  |
| A causa de tu trabajo ¿Te notas irritado/a? |  |  |  |
| ¿Te cuesta concentrarte en otras actividades, por estar pensando en cosas relacionadas al trabajo? |  |  |  |
| A causa de tu trabajo ¿Tomas tranquilizantes? |  |  |  |
| A causa de tu trabajo ¿Sientes dolor de la cuello, espalda o cintura? |  |  |  |
| A causa de tu trabajo ¿Sientes molestias oculares (deslumbramiento y/o parpadeo)? |  |  |  |

**Final version of the 25-item questionnaire**

| Initial item | **Survey: Perception of Dentists on Occupational Health and Safety** | Final item |
| --- | --- | --- |
|  | **F1: Work Demands and Well-being** |  |
| 45 | From the perspective of nervous fatigue, do you consider your usual work pace to be appropriate? | 1 |
| 47 | Does your job allow you to sleep well at night? | 2 |
| 48 | Do you think the recovery from fatigue between one workday and the next is sufficient? | 3 |
| 58 | Are the number and total duration of breaks during the workday sufficient? | 4 |
| 61 | Can you choose your schedule? | 5 |
| 62 | Do you consider the distribution of your work schedule to be adequate? | 6 |
| 64 | Can you leave your work for a few minutes without needing a replacement? | 7 |
| 66 | Can you vary your work pace without disrupting production throughout the day? | 8 |
|  | **F2: Ergonomics and physical conditions of the environment** |  |
| 2 | Are the cables of dental equipment (dental unit, compressor, oven, X-ray equipment, etc.) and/or plugs properly protected? | 9 |
| 7 | If the instruments are electrical, do they have the proper protection? | 10 |
| 17 | Is the lighting positioned in a way that prevents glare and reflections? | 11 |
| 24 | Do you consider the temperature in your workplace to be appropriate? | 12 |
| 37 | Are the work areas, changing rooms, restrooms, and cafeterias kept in perfect cleaning and disinfection conditions? | 13 |
| 51 | Is your seat comfortable? | 14 |
| 53 | Do you have enough space to change the position of your legs and knees? | 15 |
| 56 | In general, do you have enough space to perform your work comfortably? | 16 |
|  | **F3: Safety and risk prevention** |  |
| 12 | Are there warning signs and/or hazard alerts in your workplace? | 17 |
| 13 | Does your workplace have fire-fighting equipment, such as a fire hose and extinguishers? | 18 |
| 15 | Are there regulations in your workplace regarding the handling and transportation of dental materials and supplies? | 19 |
| 16 | Are regular inspections and maintenance performed on the dental equipment? | 20 |
| 79 | Does your immediate supervisor ask for your opinion on decisions that affect the work you do? | 21 |
|  | **F4: Working conditions and worker protection** |  |
| 32 | Are there proper environments for handling dental chemical supplies (workplace)? | 22 |
| 91 | Based on the work you do; do you know what benefits you are entitled to? | 23 |
| 97 | Are there people at your workplace trained in first aid to provide assistance? | 24 |
| 106 | Are there posters at your workplace indicating the mandatory use of PPE? | 25 |

**Spanish version**

| No. de pregunta inicial | **Cuestionario: Percepción de odontólogos sobre seguridad y salud en el trabajo** | No. de pregunta final |
| --- | --- | --- |
|  | **F1: Exigencias laborales y bienestar** |  |
| 45 | Desde el punto de vista de la fatiga nerviosa ¿Consideras que tu ritmo habitual de trabajo es adecuado? | 1 |
| 47 | ¿Tu trabajo te permite dormir bien por las noches? | 2 |
| 48 | ¿Crees que la recuperación de la fatiga entre una jornada de trabajo y la siguientes es suficiente? | 3 |
| 58 | ¿El número y la duración total de las pausas durante la jornada laboral, son suficientes? | 4 |
| 61 | ¿Puedes escoger tu horario? | 5 |
| 62 | ¿Consideras adecuada la distribución del horario de trabajo? | 6 |
| 64 | ¿Puedes abandonar tu trabajo por unos minutos sin necesidad de que te sustituyan? | 7 |
| 66 | ¿Puedes variar tu ritmo de trabajo sin perturbar la producción a lo largo de la jornada? | 8 |
|  | **F2: Ergonomía y condiciones físicas del entorno** |  |
| 2 | ¿Los cables de los equipos odontológicos (unidad dental, compresora, estufa, equipo de Rx, etc) y/o enchufes están protegidos? | 9 |
| 7 | ¿Si el instrumental es eléctrico tienen la protección adecuada? | 10 |
| 17 | ¿La luz está situada de forma que impida deslumbramientos y reflejos? | 11 |
| 24 | ¿Consideras que la temperatura de tu lugar trabajo es la adecuada? | 12 |
| 37 | ¿Se mantienen las zonas de trabajo, vestuario, aseo y comedores en perfectas condiciones de limpieza y desinfección? | 13 |
| 51 | ¿Tu asiento es cómodo? | 14 |
| 53 | ¿Tienes espacio suficiente para variar la posición de piernas y rodillas? | 15 |
| 56 | En general ¿Dispones de espacio suficiente para realizar el trabajo con holgura? | 16 |
|  | **F3:** **Seguridad y prevención de riesgos** |  |
| 12 | ¿Existen señales de atención y/o advertencias frente al peligro en tu lugar de trabajo? | 17 |
| 13 | ¿Cuenta tu lugar de trabajo con equipos contra incendios, por ejemplo, manguera para bomberos y extintores? | 18 |
| 15 | ¿Existen normas dictadas en tu lugar de trabajo respecto a la manipulación y transporte de materiales e insumos odontológicos? | 19 |
| 16 | ¿Se realizan revisiones y mantenimiento periódico de los equipos odontológicos? | 20 |
| 79 | ¿Tu jefe inmediato te pide opinión en las decisiones que afectan el trabajo que realizas? | 21 |
|  | **F4:** **Condiciones laborales y protección del trabajador** |  |
| 32 | ¿Existen ambientes adecuados para el manejo de insumos químicos odontológicos? | 22 |
| 91 | Según el trabajo que realizas ¿Sabes a qué prestaciones tienes derecho? | 23 |
| 97 | ¿Hay personas que puedan prestar los primeros auxilios con la formación de socorristas? | 24 |
| 106 | ¿En tu lugar de trabajo hay afiches que indiquen el uso obligatorio de EPP? | 25 |
